# Hypertensive diabetic patients: incidence of cardiovascular and renal outcomes in a historical cohort over 11 years

**DOI:** 10.1186/s13098-017-0296-z

**Published:** 2017-12-21

**Authors:** Andréa Cristina Sousa, Thiago Veiga Jardim, Thiago Olivera Costa, Fabrício Galdino Magalhães, Marcos Paulo Marinho Montelo, Weimar K. Barroso Souza, Paulo César Brandão Veiga Jardim, Ana Luiza Lima Sousa

**Affiliations:** 1 Nursing School, Federal University of Goiás (Universidade Federal de Goiás), Goiânia, Brazil; 2 Hypertension League, Federal University of Goiás (Universidade Federal de Goiás), 1ª Avenida, S/N-Setor Leste Universitário, Goiânia, GO 74605-020 Brazil; 3 Medical School, Federal University of Goiás (Universidade Federal de Goiás), Goiânia, Brazil

**Keywords:** Hypertension, Type 2 diabetes mellitus, Cardiovascular diseases, Diabetes complication

## Abstract

**Background:**

Diabetics have increased risks for cardiovascular disease (CVD) and mortality, reducing their life expectancy by up to 15 years. Type 2 diabetes mellitus specifically increases the risk for cardiovascular mortality nearly fivefold. When hypertension is combined with diabetes, the risk of CVD is even greater.

**Objective:**

Identify non-fatal cardiovascular outcomes and renal function impairment in a cohort of hypertensive patients in regular treatment in a reference treatment center, over 11 years of follow-up.

**Methods:**

Historical cohort of hypertensive patients in regular treatment for at least 11 years in a specialized service for hypertension treatment. The exposed group was hypertensive diabetic patients at the beginning of the cohort, and the non-exposed group had hypertension without diabetes. The cohort began in 2004, with follow-ups in 2009 and 2015. Variables used: gender, race, age, physical activity, alcohol consumption, smoking, blood pressure, body mass index, glycated hemoglobin, diabetes and hypertension diagnosis times, treatment time in specialized service, non-fatal cardiovascular outcomes, and renal impairment assessed by creatinine clearance.

**Results:**

139 patients participated in the study (55 diabetic hypertensive; 84 non-diabetic hypertensive), with an initial (2004) mean hypertension treatment time of 5.8 years. Females were the majority (75.5%) in both groups. Groups were similar regarding socio-demographic variables, but the group of hypertensive diabetic patients had higher frequency of obesity and uncontrolled BP, which persisted in all follow-ups. In 11 years of follow-up (2004–2015), the diabetic group had more cardiovascular events, with increased risk of acute myocardial infarction (RR 95% CI 1.6 12.2–95.0), stroke (RR 95% CI 1.3–6.1 27.7) and complications requiring hospitalization (RR 95% CI 1.6 2.2–3.0). Worsened renal function occurred more often in the non-exposed group, but in the end, the proportion of renal function loss was similar between groups.

**Conclusions:**

Exposure to type 2 diabetes increased the risk of new cardiovascular outcomes over 11 years of follow-up of hypertensive patients. Diabetes by itself increased the risk of cardiovascular outcomes, justifying more intensive actions in this population.

## Background

Hypertension (HTN) is a well-established risk factor for cardiovascular diseases in people with diabetes [1]. On the other hand, type 2 diabetes (T2DM) is an independent risk factor for cardiovascular diseases such as atherosclerosis and heart failure [2]. T2DM increases the risk of cardiovascular mortality by 4.9 times, with a life expectancy reduction ranging from 5 to 15 years, depending on the age at diagnosis [3, 4]. When T2DM is associated with HTN, the risk of developing CVD becomes even greater [5]. Values of systolic blood pressure (SBP) ≥ 140 mmHg in patients with T2DM increase the risks of coronary heart disease, stroke, cardiovascular events and mortality from all causes [6].

Many studies on the cardiovascular risk factors associated with T2DM have been conducted outside Brazil. Studies to identify these associations require long-term follow-up and are difficult to be held because of its high costs. The possibility of retrospective analysis imposes the need for records of data from the same population over a long period of time. Information about health conditions of the population and their demands for medical and social services are fundamental for health care planning and health promotion. Health conditions and outcomes of the population are of great relevance to identify better ways of approaching their health issues.

This study evaluated whether hypertensive patients exposed to T2DM had increased risk of new non-fatal outcomes such as stroke, acute myocardial infarction, myocardial revascularization, acute coronary syndrome, renal impairment and hospitalizations, despite regular treatment in a specialized care unit, with a follow-up protocol implemented in 1989.

## Methods

This was a non-concurrent historical cohort study that included hypertensive diabetic and non-diabetic patients with regular outpatient follow-up (at least 11 years, counted retrospectively) in a clinic specialized in hypertensive patients’ treatment in the midwest region of Brazil. We excluded those diagnosed with chronic kidney disease and those who developed T2DM between 2004 and 2015, in order to avoid bias from diabetes exposure.

**Fig. 1:**
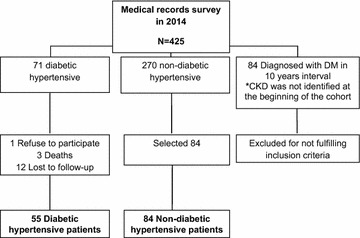
Cohort inclusion flow chart

The exposed group was composed of hypertensive patients who were diabetic in 2004, and the non-exposed group was selected randomly to number 1.5 times the exposed group (Fig. 1).

The study protocol was submitted and approved by the Ethics Committee of the institution (Protocol Number 931.503). All subjects who agreed to participate signed a consent form.

The cohort had a duration of 11 years retrospectively from 2015 and was assessed in three follow-ups: initial (2004), intermediate (2009) and final (2015). The data used were those from the first consultation held in the year of each evaluation.

Sociodemographic variables were evaluated along with clinical data: HTN and T2DM diagnosis times, treatment time at the specialized service, blood pressure values, glycated hemoglobin (hemoglobin A1C—HbA1C), lipid profile and body mass index (BMI), and use of lipid-lowering and blood pressure control therapies.

The initial (2004) and intermediate follow-up (2009) BP values were those registered in medical records. For the final follow-up (2015), blood pressure was measured by the leading researcher in the office using the OMRON HEM-705CP semi-automatic device. At that time, the informed consent was signed. BP was considered controlled in the non-exposed group (non-diabetic) at values < 140/90 mmHg and the exposed group (diabetic) at values < 130/80 mmHg [7], according to the Brazilian guidelines.

For the first and second laboratory evaluations, we used the data of laboratory tests recorded in the medical record, and in the final follow-up, blood samples were collected for biochemical analysis. The reference values were creatinine < 1.3 mg/dL, lipid profile (total cholesterol < 200 mg/dL; LDL cholesterol < 100 mg/dL; HDL cholesterol > 60 mg/dL) [8], glucose (≤ 130 mg/dL for diabetic hypertensives and < 110 mg/dL for the general population), and HbA1C (≤ 7%) [9].

The classification used for the body mass index was (a) underweight (BMI < 18.5 kg/m^2^); (b) normal (BMI = 18.5–25 kg/m^2^); (c) overweight (BMI = 25–29.9 kg/m^2^); (d) obesity (BMI ≥ 30 kg/m^2^) [10].

Cardiovascular outcomes were defined as non-fatal myocardial infarction (MI), myocardial revascularization, stroke, acute coronary syndrome, hospitalizations and blood pressure uncontrolled. Fatal events were not considered outcomes because this was a non-concurrent cohort, whose composition was determined by whether patients were in regular treatment in the base year (2015). Medical records were analyzed to identify cardiovascular outcomes that occurred between the first and the second evaluation (2004–2009). In the last evaluation (2015), the participants visited the office and were asked about the occurrence of events. At this time, the medical records were evaluated again to verify the reliability of the information provided by patients.

For evaluation of renal impairment, we used the creatinine clearance, determined by the Cockcroft–Gault equation. For purposes of analysis, the estimation of glomerular filtration rate (GFR) was used considering the following categories: normal ≥ 90 mL/min/1.73 m^2^, mild kidney failure 60–89 mL/min/1.73 m^2^, moderate kidney failure 30–59 mL/min/1.73 m^2^, severe kidney failure 15–29 mL/min/1.73 m^2^ and terminal renal insufficiency (CRI) < 15 mL/min/1.73 m^2^. In addition to that, these categories were dichotomized with a cutoff point of > 60 mL/min/1.73 m^2^ for normal kidney function or mild damage, and below this value was considered moderate, severe or chronic renal failure [11].

### Statistical analysis

The qualitative variables are presented with their absolute frequencies and proportions. The associations between them were measured with the Chi square test or Fisher’s exact test, as needed. Quantitative variables are presented with their means, standard deviation, median and confidence interval. Means were compared with Student’s T test only after analysis of data distribution with the Kolmogorov–Smirnov test.

The relative risk of each outcome conferred by exposure to T2DM was calculated in the form of a 95% confidence interval. Adjusted analysis was performed for all predictors, with a significance level of < 0.20 in bivariate analysis, considering each outcome as dependent variable.

All tests were considered at a significance level of 5% and a confidence interval of 95%.

## Results

We selected 71 diabetics (exposed group) and 84 non-diabetic hypertensive patients (non-exposed group) for study participation. Among the diabetics, 12 were excluded due to follow-up loss, since they did not attend their appointments in the final follow-up. Thus, in 2015, 139 patients agreed to participate in the study. Among them, 55 were hypertensive diabetics, and 84 were hypertensive non-diabetics. At the beginning of the cohort, the mean age in both groups was similar (57.5 ± 9.3 years). The antihypertensive treatment time in the service was also the same in the two groups (5.9 ± 3.9 years). However, the average time from diagnosis of hypertension was significantly different (16.5 ± 9.5 years for the diabetic group vs. 12.2 ± 7.32 years for non-diabetics; p < 0.05). The average time from diagnosis of DM was 4.0 ± 6.0 years. The groups at the beginning of the cohort (2004) were similar regarding socio-demographic variables, except the group of hypertensive diabetic patients had higher frequencies of obesity (45.5%) and uncontrolled blood pressure (76.4%) (Table 1).

**Table 1:** Composition of non-concurrent cohort regarding socio-demographic data, clinical data and lifestyle habits, Goiânia, GO, Brazil, 2004

	Diabetic hypertensive (n = 55)		Non-diabetic hypertensive (n = 84)		Total		p*
	n	%	n	%	n	%	
Sex							0.420
Male	11	20.0	23	27.4	34	24.5	
Female	44	80.0	61	72.6	105	75.5	
Ethnicity							0.857
White	30	57.7	42	54.5	72	55.8	
Non-white	22	42.3	35	45.5	57	44.2	
No record	3	5.5	7	8.3	10	7.2	
Age (years)							0.804
< 50	12	21.8	22	26.2	34	24.5	
50–60	22	40.0	30	35.7	52	37.4	
60–70	17	30.9	23	27.4	40	28.8	
> 70	4	7.3	9	10.7	13	9.4	
Physical activity							0.488
Regular	30	55.6	47	56.0	77	55.8	
Irregular	5	9.3	13	15.5	18	13.0	
Absent	19	35.2	24	28.6	43	31.2	
No record	1	1.8	0	0.0	1	0.7	
Smoking							0.430
Yes	1	1.8	5	6.0	7	4.5	
Former	21	38.2	27	32.1	48	34.5	
Never	33	60.0	52	61.9	85	61.2	
Alcohol consumption							0.107
Yes	1	1.8	7	8.3	8	5.8	
No	54	98.2	77	91.7	131	94.2	
Number of antihypertensive drugs							0.811
0–1	17	30.9	24	28.6	41	29.5	
2–3	37	67.3	57	67.9	94	67.6	
4+	1	1.8	3	3.6	4	2.9	
Blood pressure control^a^							*<* *0.001*
Yes	13	23.6	48	57.1	61	43.9	
No	42	76.4	36	42.9	78	56.1	
Lipid-lowering agent use							0.302
Yes	6	10.9	3	3.6	9	6.5	
No	49	89.1	81	96.4	130	93.5	
Body mass index							*0.003*
Normal	9	16.4	27	32.1	36	25.9	
Overweight	21	38.2	41	48.8	62	44.6	
Obese	25	45.5	16	19.0	41	29.5	
Glycemic control^b^							–
Yes	9	19.1	–	–	9	19.1	
No	38	80.9	–	–	38	80.9	

* Pearson’s Chi square

^a^Blood pressure parameters: diabetic hypertensive < 130/80 mmHg and non-diabetic hypertensive < 140/90 mmHg

^b^HbA1C control ≤ 7%

In 11 years of follow-up, both groups decreased diastolic blood pressure, total cholesterol, low-density lipoproteins (LDL), triglycerides and creatinine clearance. Among the hypertensive diabetics in the same period, there was a significant increase in high-density lipoproteins (HDL) and a reduction in triglycerides (Table 2).

**Table 2:** Initial and final follow-up comparison of non-concurrent cohort according to groups (hypertensive diabetics and non-diabetics) in laboratory and clinical parameters, Goiânia, GO, Brazil, 2004–2015

	Diabetic hypertensive (n = 55)			Non-diabetic hypertensive (n = 84)		
	2004	2015	p**	2004	2015	p**
	Median	Median		Median	Median	
	(95% CI)	(95% CI)		(95% CI)	(95% CI)	
SBP	139.0 (132.0–143.0)	134.0 (133.0–143.8)	0.586	130.7 (129.4–138.2)	131.0 (129.5–136.8)	0.742
DBP	85.0 (81.9–89.8)	73.0 (71.8–77.8)	*<* *0.001*	82.0 (81.7–87.4)	73.7 (71.8–76.3)	*<* *0.001*
BMI	29.3 (29.5–32.5)	29.3 (28.9–32.1)	0.080	26.3 (25.8–27.6)	27.1 (26.1–28.1)	0.081
GFR	139.0 (136.1–167.5)	139.0 (129.8–167.5)	0.891	96.0 (92.2–99.5)	93.0 (91.5–98.1)	0.655
TC	215.0 (200.9–229.8)	165.0 (157.2–184.8)	*<* *0.001*	201.5 (198.3–218.1)	181.6 (175.4–188.8)	*<* *0.001*
HDL	47.0 (43.6–51.5)	51.0 (49.2–56.2)	*0.009*	47.0 (45.0–50.3)	48.0 (45.7–51.2)	*0.056*
LDL	126.0 (111.5–197.3)	85.3 (80.4–95.3)	*<* *0.001*	129.5 (125.3–141.2)	112.0 (103.2–115.1)	*<* *0.001*
TG	179.0 (169.4–257.1)	134.0 (117.3–249.8)	*0.024*	110.5 (119.5–163.7)	114.0 (113.9–140.9)	0.825
CC	82.9 (76.7–87.7)	64.2 (62.2–79.1)	*<* *0.001*	70.2 (70.5–80.2)	61.9 (60.3–71.7)	*<* *0.001*
HbA1c	9.2 (5.0–15.0)	7.4 (5.5–13.3)	*0.002*	–	–	–

*BMI* body mass index, *SBP* systolic blood pressure, *DBP* diastolic blood pressure, *GFR* glomerular filtration rate, *TC* total cholesterol, *HDL* HDL cholesterol, *LDL* LDL cholesterol, *TG* triglycerides, *CC* creatinine clearance, *HbA1c* hemoglobin, *95% CI* 95% confidence interval

** Mann–Whitney U test

In the final evaluation of this cohort, 43.6% of hypertensive diabetics showed renal impairment (creatinine clearance < 60/min/1.73 m ^2^) and 45.2% of the non-diabetic group; in other words, between the initial and final follow-up, the proportion of renal impairment increased significantly in both groups, but they stayed similar to each other (Table 3).

**Table 3:** Outcomes of 11 years in a historical cohort between diabetic hypertensive and non-diabetic hypertensive patients, Goiânia, GO, Brazil, 2015

Total outcomes	11 years					
	Diabetic hypertensive		Non-diabetic hypertensive		p*	RR
	n	%	n	%		IC 95%
Uncontrolled blood pressure	39	70.9	26	31.0	*<* *0.001*	2.3 (1.6–3.3)
AMI	8	14.5	1	1.2	*0.002*	12.2 (1.6–95)
Stroke	8	14.5	2	3.7	*0.007*	6.1 (1.3–27.7)
Myocardial revascularization	20	36.4	25	29.7	0.417	1.2 (0.7–1.9)
Acute coronary syndrome	15	27.3	23	27.4	0.988	1.0 (0.6–1.7)
Hospital admissions	44	80.0	31	36.9	*<* *0.001*	2.2 (1.6–3)
Renal impairment	23	43.4	38	45.2	0.692	0.9 (0.6–1.4)

*AMI* acute myocardial infarction

* Chi square test

**Fig. 2:**
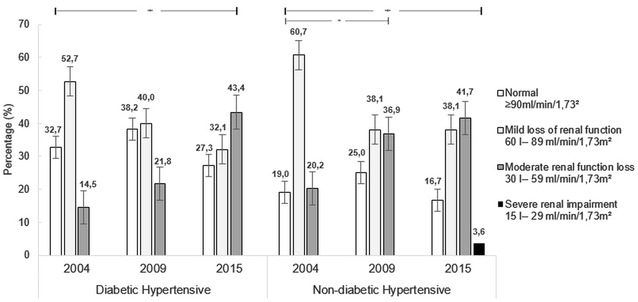
Distribution of patients with different categories of renal function throughout the study period (2004–2015), Goiânia, GO, Brazil, 2004–2015. McNemar–Browker test: *p < 0.05

Renal function, assessed by the proportion of patients in the different categories, suffered significant changes over the follow-up course. There was a greater rate of renal impairment among the non-diabetic hypertensive group. This group initially showed 60.7% of participants with mild renal lesion (60–89 mL/min/1.73 m^2^), and 19.0% could be considered within the range of normal (≥ 90 mL/min/1.73 m^2^); in the diabetic group, the mild impairment was 52.7%, and 32.7% had rates within the standards of normality. Both groups had increases in the frequency of those with moderate renal impairment (30–59 mL/min/1.73 m^2^) over the 11 years of follow-up (Fig. 2).

Regarding cardiovascular outcomes, in the intermediate evaluation, both groups showed similar occurrences of AMI, acute coronary syndrome, myocardial revascularization and hospitalization. The proportion of hospitalizations in each group at the intermediate evaluation surpassed 20%. However, the group of hypertensive diabetics had a higher incidence of stroke (exposed: n = 5, 9.1%; non-exposed: n = 1, 1.2%), with a relative risk of 7.6, counting the period of 5 years of follow-up from 2004 (Table 3).

Regarding blood pressure control, diabetics showed lower rates of control throughout the entire cohort (23.6, 27.3, and 29.1%, respectively) when compared to the unexposed group (57.1, 67.9, and 69.0%) in the three follow-ups (p < 0.05). The additional risk presented by the diabetic group was 2.3 times higher than in the other group on the intermediate and final follow-ups (p < 0.001).

Overall, in 11 years of follow-up, the group of hypertensive diabetic patients presented more cardiovascular events and a higher risk of cardiovascular events: the risk of AMI was 12.2 times greater, the risk of stroke was 6.1 times greater, and the risk of hospital admissions was 2.2 times higher compared to non-diabetics (Table 3). In the adjusted analysis, no predictive variable was associated with the outcomes analyzed. The diagnosis time of T2DM and treatment in the service were not associated with the frequency of events.

Diabetic hypertensive patients had more cardiovascular complications and hospitalizations compared to non-diabetic hypertensive patients. The main non-fatal outcomes found in our study were stroke, AMI, hospitalizations and uncontrolled blood pressure, with the risk of hypertensive diabetics raised in relation to the non-diabetics.

## Discussion

Our service is constituted in the Brazilian public health care system and is based on the attendance of a hypertensive population, aiming at pressure control and consequently the prevention of cardiovascular outcomes, since diabetes mellitus and arterial hypertension cause diseases that entail high costs, both socially and economically. A multiprofessional team (doctors of various specialties, nurses and nutritionists) performs our services, but even so, this study showed a higher incidence and risk of AMI, stroke, hospitalization and uncontrolled BP among hypertensive diabetics. Type 1 or 2 DM patients have CVD rates 4–10 times higher than non-diabetic individuals [12]. The occurrence of these outcomes is likely related to the interaction between the side effects of hyperglycemia in association with other risk factors, such as hypertension, dyslipidemia and obesity [13].

Diabetics who are even being treated for HAS and dyslipidemia still have an increased risk of CVD, showing at least twice the risk of stroke and a death risk approximately 20% higher compared to non-diabetic individuals [14]. The relative risk for stroke in this study was six times higher among diabetic individuals. A cohort study in England, with duration of 5.5 years in 1,900,000 people, showed that diabetics had a higher risk of ischemic stroke [15]. The same situation was identified in a cohort study (17 years) of 10,582 Japanese, where the risk of non-embolic ischemic stroke was approximately two times higher in individuals with type 2 DM than in non-diabetic individuals [16]. A meta-analysis involving 102 prospective studies demonstrated that the adjusted risk rate for ischemic stroke was two times higher in diabetic individuals [17]. We must consider that the study sample consisted of hypertensive patients with an average age above 60 years in the final analysis.

The time of DM exposure is also considered a risk factor for stroke, indicating that each year of disease diagnosis increases the outcome risk by up to 3% [18]. In this study, there was no association between duration of DM diagnosis and any outcome. We should emphasize that our patients were under treatment in a specialized service, and the study sample was homogeneous.

In 11 years of follow-up, the exposed group presented 12.2-fold higher risk to be affected by AMI. In the English cohort study mentioned above, non-fatal AMI risk was 1.5 times greater for the hypertensive diabetics [15]. Again our study found rates higher than those reported in the literature and that may be related to the socio-demographic specificities of the study population from which the sample was selected. Clinical trials have shown that simply improving glycemic control is unlikely to prevent the increased mortality after AMI in patients with DM, because intensive glucose control cannot reduce cardiovascular events, as well as the mortality rate in patients with DM [19]. The control of other risk factors, such as dyslipidemias, is considered more effective than glucose-lowering therapy to reduce the incidence of cardiovascular events.

Diabetics in this study showed bad glycemic control, although it improved over the follow-up time (16.4% controlled initially vs 41.8% at the end of follow-up). On the other hand, they showed worse BP control rates. It should be noted that stricter parameters were considered for BP control among diabetics [20]. T2DM patient care should be the result of integral management of cardiovascular risk, which includes other factors than glucose, such as dyslipidemias, obesity and hypertension [3].

We observed a higher rate of hospitalization among hypertensive diabetics. A study evaluating the independent contributions of HbA1c, systolic blood pressure and LDL cholesterol control to hospitalizations in patients with T2DM demonstrated that patients without control of these risk factors or with just the HbA1c controlled showed the highest rates of cardiovascular hospitalization, while those with all risk factors controlled had the lowest rates. This same study showed that to keep SBP < 130 mmHg or LDL-C < 100 mg/dL was significantly associated with reducing the risk of cardiovascular hospitalization, but keeping HbA1c < 7% did not translate to a reduction in CVD hospitalization risk [21]. Another study comparing rates of cardiovascular complications that required hospital admission among diabetic individuals from various countries revealed that European patients had a rate of 8.4%, patients in South Asia had 6.0% and Chinese patients had 3.8% [22].

DM is in itself an independent risk factor for coronary artery disease, and the risk is dramatically higher when hypertension is a comorbidity [23]. In this study, the diabetic patients had their best BP pressure control rate of 29.1% in the last follow-up. HTN affects approximately 70% of patients with diabetes and is approximately twice as common in people with diabetes [24]; it significantly increases the risk of vascular complications, predisposing these patients to chronic kidney disease (CKD) [25] and substantially increasing the risk of ischemic stroke [26].

There are still some uncertainties and controversies over the BP reduction target in hypertensive patients, especially those above 60 years, who typically feature higher levels of systolic blood pressure [27]. Recently, the SPRINT study concluded that among hypertensive patients at high cardiovascular risk, but without diabetes, treatment aiming for a systolic blood pressure of less than 120 mmHg, compared with blood pressure less than 140 mmHg, resulted in lower rates of cardiovascular events and death [28], i.e., the lower the goals for blood pressure control, the greater the benefits to be achieved in a population of non-diabetics hypertensive.

Regarding diabetic hypertensive, BP goals have been widely discussed, since there is no consensus if the same goal of BP < 140/90 mmHg should be applied. In the ACCORD study, with a population of hypertensive diabetics, intensive BP and glucose control was tested, and it demonstrated a reduction in the number of isolated strokes compared with controlling BP to a lower value (120 mmHg). On the other hand, the number of serious adverse events increased as a result of the intensive treatment of hypertension and glycemia [29].

After the publication of the SPRINT study, in 2015, the researchers of the ACCORD study supported the position of the benefits of intensive reduction of systolic BP to < 120 mmHg in patients with high cardiovascular risk and that these goals should also be extended to hypertensive diabetics, though they acknowledged some limitations in their study of intensive control of blood glucose and BP [30].

In the present study, the hypertensive patients without a diabetes diagnosis showed a worse evolution of renal function. HTN is the most important independent risk factor for renal involvement that is associated with increased cardiovascular morbidity and mortality [31]. Chronic kidney disease is the 18th cause of overall mortality [32]. In hypertensives, chronic kidney disease (CKD) is less frequent than among diabetic patients [33], but it is also considered harmful to the prognosis of the disease [34]. Treating hypertension improves prognosis, as it slows the progression of kidney disease [35]. Among T2DM patients, CKD occurs in 25–40% of patients [36], representing great morbidity and mortality, and is considered one of the criteria that defines a patient at greater risk of suffering future coronary events [37]. Hypertension, diabetes and dyslipidemia control are decisive in renal disease of diabetic and non-diabetic patients, aiming to reduce cardiovascular events as well [38].

## Limitations

Because this is a retrospective study and the criteria for inclusion were to be in regular treatment in our service with an active chart, it was not possible to identify the casualties that may have occurred between the years of 2004–2015. However, that does not minimize the results of the study, since the objective was to compare the occurrence of nonfatal outcomes in hypertensive diabetic patients to those who were hypertensive but without the development of other morbidity.

Another point to consider is that it has not been possible to analyze the correlations between the values of blood pressure and the incidence of outcomes, because we used chart registry data that did not allow the identification of the BP values at the outcome time. This is an intrinsic limitation to non-concurrent cohort studies, and only prospective studies would permit this assessment. The cohort of participants in this study should remain under evaluation, with regular follow-ups that will allow for assessing such results in the future.

## Conclusions

Both groups showed cardiovascular outcomes, as well as hospitalizations and renal impairment. However, an increased frequency of these outcomes was found among the diabetic patients. Hypertensive diabetics, as a result of metabolic and vascular damage, are more prone to cardiovascular events, even when the risk factors are treated early and followed.

The diabetic hypertensive group had 12 times the risk of AMI, more than six times the risk of stroke, and more than twice the risk of hospitalization as the non-exposed group. Therefore, more intensive actions are needed in this population to reduce the morbidity and the mortality caused by the presence of these co-morbidities.

Decreased renal function occurred more often in the hypertensive group without diabetes, even though in the exposed group, we also identified a high rate of renal impairment.

## Abbreviations

mmHg: millimeters of mercury
T2DM: type 2 diabetes mellitus
BP: blood pressure
DBP: diastolic blood pressure
SBP: systolic blood pressure
BMI: body mass index
CI: confidence interval
CKD: chronic kidney disease
HTN: hypertension
CVD: cardiovascular disease
MI: non-fatal myocardial infarction
CRI: terminal renal insufficiency
LDL: low density lipoproteins
HDL: high-density lipoproteins
GFR: glomerular filtration rate
TC: total cholesterol
TG: triglycerides
CC: creatinine clearance
HbA1c: hemoglobin A1C
CHD: coronary heart disease
